# A population‐based comparison of treatment patterns, resource utilization, and costs by cancer stage for Ontario patients with triple‐negative breast cancer

**DOI:** 10.1002/cam4.3038

**Published:** 2020-08-30

**Authors:** Christine Brezden‐Masley, Kelly E. Fathers, Megan E. Coombes, Behin Pourmirza, Cloris Xue, Katarzyna J. Jerzak

**Affiliations:** ^1^ Division of Medical Oncology and Hematology Faculty of Medicine University of Toronto Mount Sinai Hospital Toronto Ontario Canada; ^2^ Department of Medical Affairs Hoffmann‐La Roche Limited Mississauga Ontario Canada; ^3^ Market Access and Pricing Department Hoffmann‐La Roche Limited Mississauga Ontario Canada; ^4^ Division of Medical Oncology and Hematology Faculty of Medicine University of Toronto Sunnybrook Odette Cancer Center Toronto Ontario Canada

**Keywords:** cohort studies, costs and cost analysis, drug therapy, health services research, radiotherapy, surgical procedures operative, triple‐negative breast neoplasms

## Abstract

**Background:**

There have been few publications exploring the characteristics, treatment pathways, and health‐care costs by stage in patients with a triple‐negative breast cancer (TNBC) phenotype.

**Methods:**

Data from a publicly funded health‐care system in Ontario were assessed. Baseline characteristics, treatment patterns, and health‐care costs were descriptively compared by cancer stage (I‐III vs IV) for adult women diagnosed with invasive TNBC between 2012 and 2016. Resource use was multiplied by unit costs for publicly funded health‐care services to calculate health system‐related costs.

**Results:**

A total of 3271 cases were identified, 3081 with stage I‐III and 190 with stage IV TNBC. Baseline characteristics were aligned with previous reports. Surgery was the most common treatment among patients with stage I‐III disease (n = 2979, 96.7%); 557 (18.7%) received neoadjuvant therapy (NAT) and 1974 (66.3%) received adjuvant therapy (AT), the latter at a median of 44 days postsurgery, and 2446 (79.4%) in the stage I‐III cohort received radiation. Treatment for metastatic TNBC included surgery in 48 (25.3%), systemic therapy in 138 (72.6%), and radiotherapy in 112 (58.9%) patients. Top drug regimens included anthracyclines/taxanes. Annual per‐patient health care costs were four times higher for stage IV vs. stage I‐III TNBC.

**Conclusion:**

Per‐patient costs were higher in metastatic TNBC, despite a less frequent use of all treatment modalities compared to early TNBC. Treatment patterns were aligned with the options available at the time; however, neoadjuvant treatment rates were low.

## Graphical abstract

Characteristics and treatment patterns of Ontario patients diagnosed with TNBC are similar to that of other contemporary reports; however, it appears that uptake of neoadjuvant therapy could be improved. Cost of metastatic disease is high and novel treatments should be adopted and evaluated for their impact.

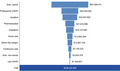

## 1 INTRODUCTION

Breast cancer (BC) is a heterogeneous disease that can be defined by morphologic or molecular features that predict a patient's prognosis. One of the most clinically driven classification schemes relies on immunohistochemical measures of hormone and human epidermal growth factor receptor 2 (HER2) receptor expression to guide systemic therapy.^1^ In this histological classification, tumors with a triple‐negative phenotype test negative for estrogen (ER), progesterone (PR), and HER2 receptors.^1^ Triple‐negative breast cancer (TNBC) represents approximately 15% of all breast carcinomas.^2^

With the majority of TNBC tumors lacking targetable receptors, chemotherapy remains the predominant treatment option for patients with TNBC.^3^ Although considered chemo‐sensitive, recurrence rates are high in the first 3 years after diagnosis and survival after metastasis is shorter compared with other BC subtypes.^3^, ^4^, ^5^

Population‐based studies focused on TNBC are sparse and the majority address only clinical characteristics and outcomes.^3^, ^5^, ^6^, ^7^, ^8^, ^9^, ^10^, ^11^, ^12^, ^13^, ^14^, ^15^, ^16^ While older data report direct health‐care costs for Ontario BC patients by cancer stage,^17^ there are no published reports on resource utilization in an Ontario TNBC cohort by cancer stage. We sought to assess the occurrence, management, and resource utilization according to early versus de novo metastatic disease in Ontario women diagnosed with TNBC.

## 2 METHODS

### 2.1 Study design

We conducted a retrospective, observational, population‐based study to assess the treatment, resource utilization, and health‐care costs for a cohort of Ontario women diagnosed with stage I‐III versus stage IV TNBC. The study was approved by the Ontario Cancer Research Ethics Board and conducted in 2019 by ICES using all relevant databases under their purview.

ICES is an independent nonprofit organization that houses de‐identified population‐based health and social data on publicly funded services provided in Ontario. Cases are linked across databases by their unique Ontario Health Insurance Plan (OHIP) number. In Ontario, all Canadian citizens and permanent residents are eligible to receive publicly funded hospital care, most physician services, outpatient and emergency services, and, for those 65 years of age or older or on social assistance, prescription drug coverage. Supplemental drug funding is also provided by the government through special programs within the Ontario Drug Benefit (ODB) program or the New Drug Funding Program (NDFP).

Incident cases of invasive BC^18^ in adult women (18‐105 years old) diagnosed between 1 April 2012 and 31 March 2016 were extracted from the Ontario Cancer Registry (OCR). Those diagnosed with a secondary non‐BC malignancy were excluded from the analysis, as well as any incomplete/invalid records (ie, missing age/gender). The final cohort only included patients with a known and negative HER2 test result, known and negative HR status, and a documented cancer stage (Figure S1). Molecular subtype was determined from synoptic pathology reports housed in the OCR. Receptor status was defined according to the Cancer Care Ontario (CCO) and ASCO/CAP guidelines.^19^, ^20^, ^21^ Patients were assigned to the stage I‐III or stage IV cohort based on their stage at initial diagnosis; therefore, all patients in the stage IV cohort had de novo metastatic disease. Patients were followed until the earliest of the following: date of last contact with the health‐care system, end of OHIP eligibility, death, or end of study, which was 31 March 2017.

### 2.2 Measures and data sources

Variables of interest for data collection included age, rurality,^22^ comorbidity index,^23^ income status,^24^ and various tumor characteristics. American Joint Committee on Cancer disease stage at diagnosis was reported according to the Collaborative Staging Methodology (v. 1.0, 2004), which incorporates TNM information.^25^ Tumor characteristics of interest were derived from the OCR and included histologic grade (reported according to the Nottingham combined scoring system),^26^ laterality, pathologic tumor size, and lymph node status.

Treatment‐related variables of interest included treatments received (surgery, radiation, or systemic therapy), time between diagnosis and the start of each treatment modality, and the top three systemic regimens commonly prescribed according to line of therapy. The authors reviewed database treatment codes and ensured queries related to systemic therapy were specific to anticancer therapies. Surgery dates and types were derived from the Canadian Institute for Health Information (CIHI) Discharge Abstract and Same Day Surgery databases. Rates of radiation therapy (RT) were calculated using radiation exposure data captured in OHIP, National Ambulatory Care Reporting System (NACRS), and/or the Cancer Activity Level Reporting (ALR) databases between diagnosis and study end. Rates of systemic therapy were calculated using drug exposure data from the OHIP, NACRS, ALR, NDFP, and/or ODB databases.

For patients with stage I‐III disease, systemic therapy was categorized as neoadjuvant (NAT, occurring before surgery) or adjuvant (AT, occurring within 24 weeks after surgery—a broad window intended to ensure the capture of systemic therapy in case of delay following locoregional therapy). First and second line therapy were defined as the first and second systemic treatment regimens, respectively, received following diagnosis of stage IV TNBC. Since the majority of systemic anticancer therapies are reimbursed (by ODB or NDFP), dispensed, and administered by the cancer clinics, the ALR database was considered the most comprehensive provincial record of cancer regimens received.

Health resource utilization measures included number of events/uses as well as length of stay where applicable and were queried in the databases outlined in Table S1. Costs were determined by multiplying the health resource utilized by the unit cost. Unit costs for emergency room visits, day hospitalizations/surgeries, and inpatient/rehabilitation stays were sourced from the CIHI and the Ontario Case Costing Initiative. Costs for biopsies, imaging, physician visits, and laboratory tests were sourced from the Ontario Ministry of Health and Long‐Term Care (MOHLTC). Health service cost components were summed to calculate the total direct cost for the full period of care. To estimate annual direct health‐care costs per‐patient, total costs over the study period were divided by the period of care and the number of patients. All reported costs were inflated to 2017 Canadian dollars using the Consumer Price Index calculator.^27^

For some reporting, costs were combined into themes, as follows.

- Continuous care = long‐term care + complex continuing care.
- Pharmaceutical (drug only) = ODB + NDFP.
- Inpatient = hospital + mental health + rehabilitation.
- Ambulatory noncancer = emergency department + dialysis clinic visits.

Hospital outpatient cost data were derived from the MOHLTC and defined as billings involving day surgery, medical day care, or clinic care related to clinic attendance, rehabilitation services, or diagnostic tests. These costs were then linked to OHIP records using a validated algorithm.^28^

### 2.3 Statistical methods

Considering the descriptive nature of our study and that there were approximately 9614 new cases of BC each year in Ontario,^29^ our sample size was fixed as the number of cases identified over the 4‐year period of the study.

Results are reported using descriptive statistics for center (mean, median) and dispersion (SD and interquartile range [IQR]) for all continuous variables. Categorical variables were summarized using counts and percentages. In accordance with ICES policies, cells with fewer than six patients and any interrelated cells were suppressed to prevent re‐identification.

## 3 RESULTS

### 3.1 Patient characteristics

Among the 34 340 women newly diagnosed with BC and meeting the criteria for inclusion, 3277 (9.5%) had TNBC. Six patients did not have a reported disease stage and were excluded from further analyses.

The mean age of women with stage I‐III TNBC was 58.8 (±14.4) years and 63.9 (±15.7) years in those with stage IV disease, with more than half of stage IV cases being 65 years of age or above. Table 1 highlights key demographic and tumor characteristics observed in the cohort of patients with staged disease (n = 3271) and compares subcohorts with stage I, II, or III versus stage IV disease at diagnosis. Patients diagnosed with stage IV TNBC were more often in lower income quintiles and a larger proportion had tumors >5 cm compared to those diagnosed with stage I‐III disease. The majority of patients with stage I‐III TNBC had tumors ≥2 cm, lymph node negative status, and tumors that were poorly differentiated.

**TABLE 1:** Baseline characteristics of Ontario patients with triple‐negative breast cancer (2012‐2016), by stage at diagnosis

	Stage I‐III (n = 3081)		Stage IV (n = 190)	
	No.	%	No.	%
Age, years				
18‐64	1975	64.1	87	45.8
65+	1106	35.9	103	54.2
Index fiscal year				
2012	748	24.3	50	26.3
2013	783	25.4	45	23.7
2014	783	25.4	41	21.6
2015	767	24.9	54	28.4
Rurality of residence				
Missing	3^a^	0.1^a^	0	0.0
No	2,716	88.2	169	88.9
Yes	362^a^	11.7^a^	21	11.1
Income quintile				
Missing	9^a^	0.3^a^	3^a^	1.6^a^
1 – Lowest	521^a^	16.9^a^	44^a^	23.2^a^
2	613	19.9	50	26.3
3	621	20.2	37	19.5
4	662	21.5	24	12.6
5 – Highest	655	21.3	32	16.8
Charlson Comorbidity Index				
Missing	2,131	69.2	134	70.5
Mean ± SD	0.57 ± 1.16		0.93 ± 1.41	
Laterality of primary				
Right	1518	49.3	92	48.4
Left	1563	50.7	94	49.5
Unspecified, one‐sided	0	0.0	3^a^	1.6^a^
Paired site	0	0.0	3^a^	1.6^a^
Tumor size				
No mass found	8^a^	0.3^a^	3^a^	1.6^a^
0 to <2 cm	986^a^	32.0^a^	20^a^	10.5^a^
2 to <5 cm	1625	52.7	67	35.3
5 cm or greater	430	14.0	71	37.4
Other^b^	3^a^	0.1^a^	6^a^	3.2^a^
Unknown	26	0.8	26	13.7
Lymph node status				
Negative	1928	62.6	9	4.7
Positive	1006	32.7	76	40.0
Unknown	147	4.8	105	55.3
Tumor grade				
Grade 1	53^c^	1.7^c^	3^a^	1.6^a^
Grade 2	410^d^	13.3^d^	15^d^	7.9^d^
Grade 3	2226	72.2	70	36.8
Unknown	391^a^	12.7^a^	106^a^	55.8^a^
Disease stage				
I	912^a^	29.6^a^	0	0.0
II	1608	52.2	0	0.0
III	561^a^	18.2^a^	0	0.0
IV	0	0.0	190	100.0

Abbreviation: IQR, interquartile range.

^a^ midpoint of suppressed data range, n = ±2.

^b^ diffuse disease or Paget's disease of the nipple with no tumor.

^c^ midpoint of suppressed data range, n = ±6.

^d^ midpoint of suppressed data range, n = ±4.

Of the 3271 patients included in the analysis, 651 (19.9%) died within the timeframe of study follow‐up (median 31 months [IQR: 20‐45]). This included 493 patients with stage I‐III (16.0%) and 158 (83.2%) with stage IV disease with a median follow‐up of 32 (IQR: 21‐45) and 9 (IQR: 4‐18) months, respectively.

### 3.2 Treatment

Rates of surgery, RT, and systemic therapy were all lower in the stage IV cohort compared with stage I‐III (Table 2).

**TABLE 2:** Treatment received by Ontario patients with triple‐negative breast cancer (N = 3271), by stage at diagnosis (2012‐2016)

Treatment modality	Stage I‐III (n = 3081)		Stage IV (n = 190)	
	No.	%	No.	%
Surgery (within 1 yr of diagnosis)	2979	96.7	48	25.3
Lumpectomy^a^	1925	62.5	26	13.7
Mastectomy^a^	1020^b^	33.1^b^	22^b^	11.6^b^
Lumpectomy followed by mastectomy^a^, ^c^	14^b^	0.5^b^	3^b^	1.6^b^
Lymph node excision only	17	0.6	0	0.0
Systemic therapy	2475	80.3	138	72.6
Radiation therapy	2446	79.4	112	58.9

^a^ with or without LN excision.

^b^ midpoint of suppressed data range, n = ±2.

^c^ entry occurs on same surgery record.

In the group of patients with stage I‐III disease receiving surgery (n = 2979, 96.7%), the mean number of surgeries was 1.14 (±0.38) (Table S2). The median number of days between diagnosis and first surgery was 36 (IQR: 25‐62). Among patients treated with upfront surgery for early stage disease (n = 2422, 81.3%), 1847 (76.3%) received AT. The remaining 557 (18.7%) received NAT starting at a median of 28 days (IQR: 21‐39) after diagnosis and underwent surgery at a median of 139 days (IQR: 127‐158) after the start of NAT. Approximately 4.3% (n = 127) of patients who were treated with NAT went on to receive AT after surgery. For all patients, AT was started within a median of 44 days (IQR: 35‐57) of diagnosis. Patients undergoing surgery but not receiving systemic or RT totaled 242 (8.1%).

For patients diagnosed with stage IV TNBC, surgery was the least common treatment modality (n = 48, 25.2%) compared with any RT (n = 112, 58.9%) and systemic therapy (n = 138, 72.6%). Of those undergoing surgery, 38 (79.2%) received systemic therapy and 29 (60.4%) received RT. Of those not undergoing surgery (n = 142), 100 (70.4%) received systemic treatment at a median of 34 days (IQR: 21‐54) after diagnosis, and 83 (58.5%) received RT (Table S3).

In the adjuvant and neoadjuvant settings, the most commonly used regimens included an anthracycline and/or taxane. In the metastatic setting, single agent taxane was the most commonly used first‐line drug while capecitabine and gemcitabine‐platinum represented the most common second‐line regimens (Table S4).

### 3.3 Resource utilization and costs

**FIGURE 1:**
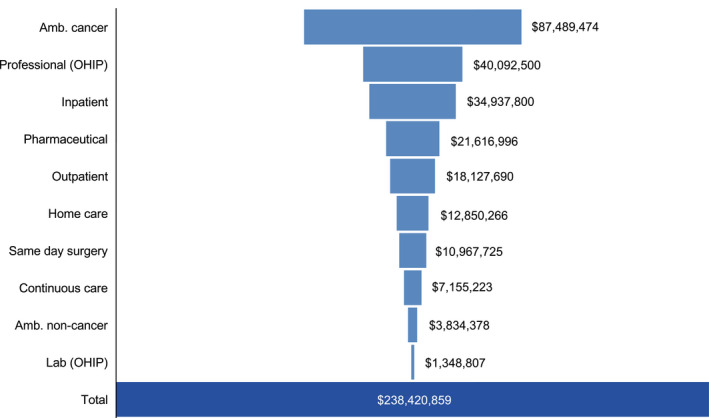
Total costs by resource type for Ontario patients with triple‐negative breast cancer (N = 3271) across study period (2012‐2017). Amb, ambulatory; OHIP, Ontario Health Insurance Plan

The full TNBC cohort was responsible for total measured costs of $238,420,859 between April 2012 and March 2017. In terms of total measured costs by resource type, ambulatory cancer clinic visits, OHIP professional fees, and inpatient hospitalizations combined accounted for over half of the costs incurred (36.7%, 16.8%, and 14.7%, respectively) (Figure 1).

For patients with stage I‐III TNBC, the average annual per‐patient total cost was $35 064, compared with $140 160 for patients with stage IV disease (Tables 3 and S5). Average annual cost for the full cohort (n = 3271) was approximately $134 662 584. Average annual per‐patient costs were higher for patients with stage IV disease for all resource use categories except same day surgery, which was higher for patients with stage I‐III disease. Outpatient cancer clinic visits, hospital inpatient services, and OHIP professional fees were the primary contributors to annual costs in both subgroups.

**TABLE 3:** Average annual per‐patient cost by resource type for patients with triple‐negative breast cancer (N = 3271), by stage at diagnosis (Ontario, 2012‐2016)

	Stage I‐III (n = 3081)		Stage IV (n = 190)	
	Cost	% of Total Cost	Cost	% of Total Cost
Total	$ 35 064	100.0	$ 140 160	100.0
Ambulatory cancer	$ 11 742	33.4	$ 24 434	17.4
Inpatient	$ 7109	20.3	$ 63 608	45.4
Professional (OHIP)	$ 5601	16.0	$ 21 379	15.3
Pharmaceutical	$ 2920	8.3	$ 4112	2.9
Outpatient	$ 2426	6.9	$ 8774	6.3
Home care	$ 1798	5.1	$ 8569	6.1
Same day surgery	$ 1505	4.3	$ 965	0.7
Continuous care	$ 1215	3.5	$ 4602	3.3
Ambulatory noncancer	$ 579	1.7	$ 3414	2.4
Lab (OHIP)	$ 170	0.5	$ 301	0.2

Abbreviation: OHIP, Ontario Health Insurance Plan.

**FIGURE 2:**
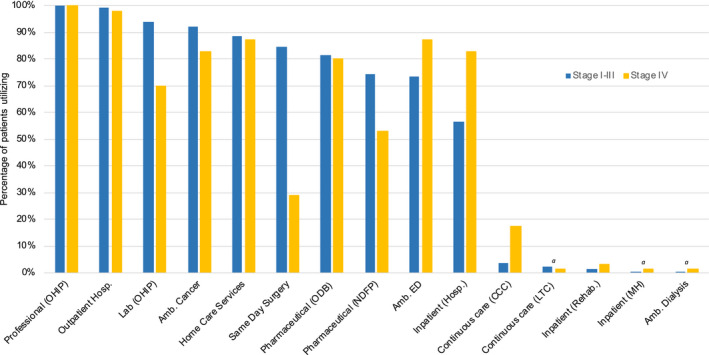
Proportion of patients with triple‐negative breast cancer, by stage, utilizing each health‐care resource (Ontario, 2012‐2017). *^a^*midpoint of suppressed data range; n = ±2. Amb., ambulatory; CCC, complex continuing care; ED, emergency; Hosp., hospital; LTC, long‐term care; MH, mental health; NDFP, New Drug Funding Program; ODB, Ontario Drug Benefit; OHIP, Ontario Health Insurance Plan; Rehab., rehabilitation

Higher utilization of inpatient, emergency, and continuous care services in the stage IV cohort (Figure 2) reflected higher per‐patient annual costs (Table 3) compared to those observed for patients with stage I‐III TNBC. Despite similar or lower utilization of professional, hospital outpatient, laboratory, ambulatory cancer clinic, home care, and long‐term care services, as well as pharmaceutical benefits (Figure 2), patients with stage IV TNBC had higher per‐patient annual costs in all of these categories compared to those with stage I‐III disease (Table 3). OHIP professional, home care, ambulatory cancer, and hospital outpatient services, as well as ODB were highly utilized by a similar proportion of patients regardless of disease stage (Figure 2; Table S5).

Of the 3271 patients with TNBC, 1898 (58%) had at least one inpatient hospital stay. Among them, patients with de novo metastatic disease had an average number of inpatient visits six times that of patients with stage I‐III disease; patients with metastatic BC also had a substantially longer average length of stay (65.1 vs 7.5 days) than patients with early BC (Table S5).

Patients with stage IV TNBC also had roughly four times the number of OHIP professional encounters and hospital outpatient visits than patients with stage I‐III disease. Costs related to home care service utilization and ambulatory cancer clinic visits were approximately quadrupled and doubled, respectively, for patients with stage IV versus stage I‐III BC (Table S5).

## 4 DISCUSSION

Patients diagnosed with de novo stage IV TNBC were more often in lower income quintiles, of older age, and had poorer prognostic tumor characteristics (higher grade and larger size). Treatments, in general, reflected the standards of care at the time of our study, except for neoadjuvant treatment rates which appeared low considering cohort characteristics. Average annual per‐patient health‐care costs were four times higher for stage IV compared with stage I‐III TNBC, mainly owing to a higher utilization and cost of inpatient services.

### 4.1 Patient characteristics

In our TNBC population, mean age of the overall cohort was comparable to reports from other population‐based studies.^5^, ^11^, ^14^, ^15^, ^16^, ^30^, ^31^ Most BCs are diagnosed at an early stage; in Canada, <5% are diagnosed at stage IV.^32^ In our study, 5.8% of TNBCs were metastatic at diagnosis, which is in line with other reports.^5^, ^11^ In our cohort, the majority of tumors were ≥2 cm (67.1%) and high grade (70.2% grade 3), which is also similar to other cohorts.^3^, ^5^, ^9^, ^13^, ^14^

### 4.2 Treatment

Surgery was the main form of treatment (received by 96%) in patients with stage I‐III TNBC. Patients underwent lumpectomy or mastectomy at rates of 63% and 33%, respectively, which demonstrates support for breast‐conserving surgery comparable to^13^ or greater than^8^, ^33^ that reported in other cohorts.

One important benchmark in early BC management is the time from diagnosis to surgery, which was found to be a median of 36 days in our study. A report from CCO highlights that in Ontario, the majority of women with BC are receiving surgical treatment within the recommended time from diagnosis, which is 28 days for invasive but not highly aggressive malignancy.^34^

The adjuvant treatment rate in our study aligns with similar contemporary cohorts^14^, ^35^ and was initiated at a median of 44 days after surgery. However, recent studies^36^, ^37^, ^38^ have shown improved outcomes in TNBC patients starting AT within 30 days, highlighting a critical opportunity for improvement. Future studies to examine factors that contribute to treatment delays in AT and barriers to NAT are warranted.

Lack of core biopsy testing was an unfortunate clinical reality for many centers in Ontario during the timeframe of our study. An absence of upfront pathological results may explain why, although ~70% of patients diagnosed with stage I‐III TNBC were stage II or III, only 20% of patients with stage I‐III BC received NAT in our study. As expected, most patients receiving NAT did not go on to receive AT given the lack of available options at the time. With new evidence to support the use of adjuvant capecitabine among patients with residual TNBC post‐NAT,^39^ an increase in the use of NAT and postoperative capecitabine is expected.^40^, ^41^

A remarkable 80% of patients with stage I‐III TNBC in our study received RT. This is similar to a Swiss study^13^ and significantly higher than other reported rates of around 48%.^7^, ^14^, ^35^ This is a positive observation considering the survival benefit of adjuvant RT seen in a Danish study^42^ and a more recent confirmative population‐based study.^35^

The rate of exposure to systemic therapy in our cohort (80% in stage I‐III and 73% in stage IV) was similar to or better than many similar populations.^3^, ^10^, ^13^, ^14^, ^30^ The top regimens observed in our TNBC population were diverse but generally included an anthracycline and/or taxane aligning with the guideline recommendations relevant at the time of our study.^43^, ^44^

Our findings reflect the clinical reality of TNBC treatment for this period (2012‐2016) when no targeted therapies were known to be of benefit and little difference was shown among traditional chemotherapy regimens. In a companion paper, we report that this cohort of stage IV TNBC patients had a particularly poor 5‐year overall survival rate of 7.4% compared to 93.3%, 78.9%, 47.2% for stage I, II, and III, respectively, further highlighting the serious unmet need in this subgroup (In Press). Recent advancements in treating patients with metastatic TNBC are encouraging, including the use of platinum or PARP inhibitors for patients with a pathogenic variant in the BRCA1/2 gene, as well as atezolizumab for patients with PD‐L1‐positive tumors.^45^, ^46^, ^47^

### 4.3 Resource utilization and costs

In the first Canadian study to look at health‐care costs in a cohort of staged TNBC patients, we have shown an even greater disparity in total annual costs for a patient with stage I‐III versus IV disease ($35 064 vs $140 160 per year, respectively) compared to other studies.^15^, ^30^ Our data underscore the high cost of treating patients with de novo metastatic TNBC, even considering the generally shorter treatment duration and a lack of more targeted and expensive biological treatments in this setting over this study period.

### 4.4 Limitations

Limitations of our study include those inherent to administrative and claims‐based data analysis, such as missing clinical variables of interest (eg, ethnicity, genetic test results, and recurrence or progression). Given our province‐wide, population‐based sampling, biases were limited, but involved the exclusion of patients without tumor staging information and the lack of prescription drug cost data for patients under 65 years of age.

Because only publicly funded services were captured in the databases utilized, a true total cost of care cannot be calculated (ie, out of pocket patient expenses, third party insurance, loss of productivity). In addition, cost data in a matched control group without TNBC were not assessed; thus, the total costs reported for these TNBC patients represent all publicly funded health‐care costs and not solely TNBC‐attributable costs.

Finally, assessing clinical outcomes or the statistical significance of differences between groups by stage at diagnosis was beyond the scope of this study, but could have provided additional context and/or highlighted implications of various treatment patterns.

## 5 CONCLUSION

The treatment approaches observed in our study reflect the lack of a single standard of care for patients with stage I‐III TNBC, but surgical, radiation, and systemic treatment rates were as high as or higher than expected. Although the use of NAT was low, we expect this to increase in the era of adjuvant capecitabine and with the availability of biomarker status on diagnostic core biopsies. There is room for improvement in time to postoperative systemic therapy among patients treated with upfront surgery.

While there were substantially fewer cases of stage IV TNBC compared with early stage disease, the resource utilization in this cohort was exponentially higher. Understanding these costs can assist in funding and policy decisions to support the evolution of care for this poor prognosis population.

Our research provides valuable descriptive data on the differences in treatment approach and resource use between patients diagnosed with stage I‐III versus stage IV TNBC, and could aid researchers in assessing the impact of innovative therapeutic strategies.

## CONFLICT OF INTEREST

KJJ has served as a consultant/speaker/advisory board attendee for Apobiologix, Esai, Genomic Health, Novartis, Pfizer, Purdue Pharma and Hoffmann‐La Roche. MEC, KEF, BP, and CX are employees of Hoffmann‐La Roche Ltd. CBM has served as a consultant/speaker/advisory board attendee for: Novartis, Hoffmann‐La Roche, Eli Lilly, Amgen, Pfizer, Astra Zeneca, Genomic Health, Merck, Taiho, Apobiologix, and Mylan. BP: conceptualization, funding acquisition, methodology, supervision, writing—original draft. KEF: conceptualization, funding acquisition, project administration, supervision, writing—original draft. CX: formal analysis, methodology, project administration. MEC: conceptualization, methodology. KJJ: data curation, methodology. CBM: data curation, methodology. All authors reviewed, edited, and approved the final manuscript.

## Supporting information

Supplementary Material

## Data Availability

Data available on request due to privacy/ethical restrictions. The data that support the findings of this study are available on request from the corresponding author. The data are not publicly available due to privacy or ethical restrictions.
