# Supplementary material for: A population‐based comparison of treatment patterns, resource utilization, and costs by cancer stage for Ontario patients with triple‐negative breast cancer
Source: Cancer Med. 2020 Aug 30;9(20):7548–57. doi: 10.1002/cam4.3038 (PMC7571809; doi:10.1002/cam4.3038)
Supplement: Supplementary file 1 — Supplementary Material [file CAM4-9-7548-s001.docx]

**SUPPORTING INFORMATION**

***A population-based comparison of treatment patterns, resource utilization and costs***

***by cancer stage for Ontario patients with triple negative breast cancer***

**FIGURE S1 CONSORT diagram of exclusions to arrive at final cohort of female cases of TNBC diagnosed and staged between Apr 1, 2012 and Mar 31, 2016 from the ICES database**

Breast cancer diagnosed between Apr 1 2012 and Mar 31 2016

N=40,929

Exclude: concurrent cancer diagnosis

N=92

Remaining cases

N=40,837

Exclude: previous cancer diagnosis

N=6,076

Remaining cases

N=34,761

Exclude: unknown sex

N=0

Remaining cases

N=34,761

Exclude: age <18 or >105

N=252

Remaining cases

N=34,509

Exclude: death date before diagnosis

N=8

Remaining cases

N=34,501

Exclude: malignant lymphomas

N=161

Remaining cases

N=34,340

Exclude: HER2 or HR status unknown

N=3.914

Remaining cases

N=30,426

Exclude: HER2 or HR positive

N=27,149

Remaining cases

N=3,277

Exclude: AJCC stage unknown

N=6

**Final Study Cohort**

**N=3,271**

Abbreviations: AJCC, American Joint Committee on Cancer; HER2, human epidermal growth factor receptor 2; HR, hormone receptor; TNBC, triple negative breast cancer.

**TABLE S1** List and description of datasets available to ICES

| **Dataset** | **Related Resource** | **Description** |
| --- | --- | --- |
| **Owner: Cancer Care Ontario** | | |
| Cancer Activity Level Reporting (ALR) *2005* | - Systemic therapy - Supportive drugs  - Radiation therapy | Contains patient level activity within the cancer system focused on radiation and systemic therapy services and outpatient oncology clinic visits. |
| **Owner: Ontario Ministry of Health and Long term Care** | | |
| Client Agency Program Enrolment (CAPE) *1999* | - Capitation costs | Registry of patients enrolled in a primary care model. Data elements include program type (family health team, family health organization, family health network, etc.) and patient enrolment status. |
| Home Care Database (HCD), Ontario Home Care Administrative System (OHCAS) *1990* | - Home care services | Captures information on all services provided or coordinated by Ontario Community Care Access Centres, including client data, intake and assessment information, admission and discharge, etc. |
| New Drug Funding Program (NDFP) *1995* | - Medication use including systemic therapy | Administered by Cancer Care Ontario, the NDFP funds new, and often very expensive, cancer drugs. |
| Ontario Cancer Registry (OCR) *1964* | - Cancer diagnosis  - Tumor characteristics | Contains the diagnosis code for invasive cancer (International Classification of Diseases version 10) and the date of diagnosis for all residents of Ontario. |
| Ontario Drug Benefit (ODB) claims *1990* | - Medication use including systemic therapy | Contains claims for oral prescription drugs covered under the ODB program. Primarily includes drug claims for individuals 65 years of age and older, but also coverage under special ODB programs. |
| Ontario Health Insurance Plan (OHIP) claims database *1991* | - Outpatient physician visits - Laboratory services - Non-physician services | Contains claims paid by OHIP, the universal, single-payer provincial health insurance plan, for services provided by all eligible health care providers, including physicians (primary and specialist), groups, and laboratories. |
| Registered Persons Database (RPDB) *1991* | - Health service subscriber data | Contains birth and death dates, age, sex, and date of last contact with health care services in Ontario. |
| **Owner: Canadian Institute for Health Information** | | |
| Continuing Care Reporting System (CCRS) *1996* | - Complex continuing care (CCC) - Long-term care (LTC) | Contains information about residents receiving facility-based continuing care services. Range of services includes CCC, extended or chronic care, and residential care providing nursing services (that is, long-term care). |
| Discharge Abstract Database (DAD) *1988* | - Inpatient hospitalizations | Contains demographic, clinical, and administrative data for inpatient hospital admissions (patient separations). |
| National Ambulatory Care Reporting System (NACRS) *2000/2003* | - Ambulatory emergency department visit, dialysis clinic visits, and cancer clinic visits | Contains data from hospital- and community-based ambulatory care services, including same day surgery, outpatient clinics, and emergency departments. |
| National Rehabilitation Reporting System (NRS) *2000* | - Rehabilitation admissions | Contains client data from adult inpatient rehabilitation facilities, such as administrative data (referral, admission, and discharge) and health and functional characteristics. |
| Ontario Mental Health Reporting System (OMHRS) *2005* | - Mental health admissions | Contains data on patients in adult designated inpatient mental health beds in acute and psychiatric facilities. Data elements include admission and discharge dates, diagnoses, service utilization, etc. |

Reference: ICES Data Dictionary [Internet]. 2019. Available from: https://datadictionary.ices.on.ca/Applications/DataDictionary/Default.aspx

| **TABLE S2** Treatments and wait times among patients with stage I-III triple negative breast cancer (Ontario, 2012–2017) | | | | | | | |
| --- | --- | --- | --- | --- | --- | --- | --- |
|  | **Variable** | **Value** |  | **Stage I-III with surgery (n=2,979)** |  | **Stage I-III without surgery (n=102)** |  |
|  | **Surgery** |  |  |  |  |  |  |
|  | No. of surgeries within 1 year of dx | Mean (SD) |  | 1.14 (0.38) |  | NA |  |
|  | No. of days between dx and surgery | Mean (SD) |  | 61 (61) |  | NA |  |
|  |  | Median (IQR) |  | 36 (25–62) |  | NA |  |
|  | **Systemic Therapy** |  |  |  |  |  |  |
|  | Patients who received | No. (%) |  |  |  | 71 (69.6) |  |
|  | Days between dx and first tx | Mean (SD) |  |  |  | 84 (114) |  |
|  |  | Median (IQR) |  |  |  | 45 (24–92) |  |
|  | **Radiation** |  |  |  |  |  |  |
|  | Patients who received | No. (%) |  | 2,383 (80.0) |  | 63 (61.8) |  |
|  | Days between dx and first tx | Mean (SD) |  | 200 (107) |  | 215 (206) |  |
|  |  | Median (IQR) |  | 194 (162–222) |  | 178 (100–235) |  |
| Abbreviations: dx, diagnosis; IQR, interquartile range; NA, not applicable; tx, treatment. | | | | | | | |

| **TABLE S3** Treatments and wait times among patients with stage IV triple negative breast cancer (Ontario, 2012–2017) | | | | | | | |
| --- | --- | --- | --- | --- | --- | --- | --- |
|  | **Variable** | **Value** |  | **Stage IV**  **with surgery**  **(n=48)** |  | **Stage IV without surgery (n=142)** |  |
|  | **Surgery** |  |  |  |  |  |  |
|  | No. of surgeries within one year of dx | Mean (SD) |  | 1.04 (0.20) |  | NA |  |
|  | No. of days between dx and surgery | Mean (SD) |  | 84 (86) |  | NA |  |
|  |  | Median (IQR) |  | 37 (25–144) |  | NA |  |
|  | Patients who received surgery only | No. (%) |  | 6 (12.5) |  | NA |  |
|  | **Systemic Therapy** |  |  |  |  |  |  |
|  | Patients who received systemic tx | No. (%) |  | 38 (79.2)  17 (35.4)*^a^* |  | 100 (70.4) |  |
|  | Days between dx and first tx | Mean (SD) |  | 33 (20)*^a^* |  | 46 (74) |  |
|  |  | Median (IQR) |  | 30 (21–38)*^a^* |  | 34 (21–54) |  |
|  | Days between first tx and surgery | Mean (SD) |  | 146 (71)*^a^* |  | NA |  |
|  |  | Median (IQR) |  | 133 (117–165)*^a^* |  | NA |  |
|  | **Radiation** |  |  |  |  |  |  |
|  | Patients who received | No. (%) |  | 29 (60.4) |  | 83 (58.5) |  |
|  | Days between dx and first tx | Mean (SD) |  | 233 (210) |  | 133 (160) |  |
|  |  | Median (IQR) |  | 171 (66–348) |  | 71 (21–191) |  |
| *^a^*in those receiving systemic tx before surgery.  Abbreviations: dx, diagnosis; IQR, interquartile range; tx, treatment. | | | | | | | |

| **TABLE S4** Top 3 chemotherapy regimens received, by treatment line and disease stage, in patients with triple negative breast cancer (Ontario, 2012–2017) | | | | | | | | | | |
| --- | --- | --- | --- | --- | --- | --- | --- | --- | --- | --- |
|  |  |  | **Stage I-III with surgery**  **(n=2,979)** | | |  | **Stage IV**  **(n=190)** | | |  |
|  |  |  | **NAT (n=507)** |  | **AT (n=1,797)** |  | **1L**  **(n=120)** |  | **2L**  **(n=74)** |  |
|  |  |  | No. (%) |  | No. (%) |  | No. (%) |  | No. (%) |  |
|  | **anthracycline and taxane** |  | 445 (87.8)*^a^* |  | 1,236 (68.8)*^a^* |  | 18 (15.0)*^b^* |  | NR |  |
|  | **taxane** |  | 61 (12.0) |  | 453 (25.2)*^a^* |  | 52 (43.3)*^a^* |  | 23 (31.1) |  |
|  | **anthracycline** |  | 46 (9.1) |  | 222 (12.4)*^b^* |  | 24 (20)*^b^* |  | NR |  |
|  | **capecitabine** |  | NR |  | NR |  | NR |  | 15 (20.3) |  |
|  | **gemcitabine and platinum** |  | NR |  | NR |  | NR |  | 14 (18.9) |  |
| *^a^*mid-point of suppressed data range, n=$\pm$4.  *^b^*mid-point of suppressed data range, n=$\pm$2.  Abbreviations: 1L, first line; 2L, second line; AT, adjuvant therapy; NAT, neoadjuvant therapy; NR, not reported as a top 3 regimen. | | | | | | | | | | |

| **TABLE S5** Number of visits, length of stay, and cost per person per year (mean ± SD) for each health care resource, by TNBC stage (Ontario, 2012-2017) | | | | | | | | | | |
| --- | --- | --- | --- | --- | --- | --- | --- | --- | --- | --- |
|  |  |  | **Full sub cohort** | | |  | **Population utilizing** | | |  |
|  | **Resource** |  | **Stage I-III**  **(n=3,081)** |  | **Stage IV**  **(n=190)** |  | **Stage I-III** |  | **Stage IV** |  |
|  | **Professional (OHIP)** | | |  |  |  | **n=3,081 (100%)** |  | **n=190 (100%)** |  |
|  | Visits (no.) |  | 46.7 ± 49.8 |  | 194.2 ± 209.1 |  | 46.7 ± 49.8 |  | 194.2 ± 209.1 |  |
|  | Cost |  | $5,601 ± 5,299 |  | $21,379 ± 21,030 |  | $5,601 ± 5,299 |  | $21,379 ± 21,030 |  |
|  | **Lab (OHIP)** |  |  |  |  |  | **n=2,893 (94%)** |  | **n=133 (70%)** |  |
|  | Visits (no.) |  | 19.6 ± 22.6 |  | 21.5 ± 48.1 |  | 20.8 ± 22.8 |  | 30.6 ± 55.0 |  |
|  | Cost |  | $170 ± 169 |  | $301 ± 528 |  | $181 ± 168 |  | $431 ± 586 |  |
|  | **Inpatient (Hosp.)** | | |  |  |  | **n=1,741 (57%)** |  | **n=157 (83%)** |  |
|  | Visits (no.) |  | 0.6 ± 1.4 |  | 5.4 ± 8.8 |  | 1.1 ± 1.7 |  | 6.5 ± 9.2 |  |
|  | LOS (days) |  | 4.2 ± 18.7 |  | 53.8 ± 89.4 |  | 7.5 ± 24.4 |  | 65.1 ± 94.6 |  |
|  | Cost |  | $6,883 ± 57,457 |  | $62,324 ± 104,160 |  | $12,180 ± 76,021 |  | $75,424 ± 110,227 |  |
|  | **Inpatient (Rehab.)** | | |  |  |  | **n=44 (1%)** |  | **n=6 (3%)** |  |
|  | Visits (no.) |  | 0.0 ± 0.1 |  | 0.1 ± 0.5 |  | 0.5 ± 0.3 |  | 2.2 ± 1.5 |  |
|  | LOS (days) |  | 0.2 ± 1.9 |  | 1.2 ± 7.9 |  | 11.7 ± 11.3 |  | 36.4 ± 28.8 |  |
|  | Cost |  | $119 ± 1,151 |  | $1,233 ± 8,084 |  | $8,342 ± 4,964 |  | $39,049 ± 26,424 |  |
|  | **Inpatient (MH)** |  |  |  |  |  | **n=16***^a^* **(0.5%)** |  | **n=3***^a^* **(1.6%)** |  |
|  | Visits (no.) |  | 0.0 ± 0.1 |  | 0.0 |  | 0.8 ± 0.4 |  | … |  |
|  | LOS (days) |  | 0.2 ± 3.1 |  | 0.1 ± 1.0 |  | 25.7 ± 31.9 |  | … |  |
|  | Cost |  | $107 ± 2,205 |  | $51 ± 697 |  | $18,288 ± 22,999 |  | … |  |
|  | **Same Day Surgery** | | |  |  |  | **n=2,600 (84%)** |  | **n=55 (29%)** |  |
|  | Visits (no.) |  | 0.6 ± 0.6 |  | 0.4 ± 1.0 |  | 0.7 ± 0.6 |  | 1.4 ± 1.3 |  |
|  | Cost |  | $1,505 ± 1,462 |  | $965 ± 3,022 |  | $1,783 ± 1,427 |  | $3,334 ± 4,891 |  |
|  | **Hospital Outpatient** | | |  |  |  | **n=3,016 (99%)** |  | **n=186 (98%)** |  |
|  | Visits (no.) |  | 7.1 ± 6.5 |  | 25.1 ± 32.9 |  | 7.1 ± 6.5 |  | 25.7 ± 33.0 |  |
|  | Cost |  | $2,426 ± 2,253 |  | $8,774 ± 11,411 |  | $2,442 ± 2,252 |  | $8,963 ± 11,460 |  |
|  | **Home Care** |  |  |  |  |  | **n=2,732 (89%)** |  | **n=166 (87%)** |  |
|  | Cost |  | $1,798 ± 3,342 |  | $8,569 ± 10,596 |  | $2,028 ± 3,483 |  | $9,807 ± 10,788 |  |
|  | **Amb. Cancer** |  |  |  |  |  | **n=2,837 (92%)** |  | **n=157 (83%)** |  |
|  | Cost |  | $11,742 ± 9,737 |  | $24,434 ± 20,326 |  | $12,752 ± 9,492 |  | $29,569 ± 18,647 |  |
|  | **Amb. Emergency** |  |  |  |  |  | **n=2,263 (73%)** |  | **n=166 (87%)** |  |
|  | Cost |  | $464 ± 835 |  | $3,413 ± 6,062 |  | $631 ± 919 |  | $3,906 ± 6,337 |  |
|  | **Amb. Dialysis** |  |  |  |  |  | **n=10***^a^* **(0.3%)** |  | **n=3***^a^* **(1.6%)** |  |
|  | Cost |  | $116 ± 2,743 |  | $1 ± 14 |  | $29,677 ± 33,905 |  | … |  |
|  | **Drug (NDFP)** |  |  |  |  |  | **n=2,288 (74%)** |  | **n=101 (53%)** |  |
|  | Cost |  | $355 ± 2,072 |  | $977 ± 2,802 |  | $478 ± 2,392 |  | $1,838 ± 3,638 |  |
|  | **Drug (ODB)** |  |  |  |  |  | **n=2,509 (81%)** |  | **n=152 (80%)** |  |
|  | Cost |  | $2,565 ± 3,900 |  | $3,138 ± 4,687 |  | $3,150 ± 4,103 |  | $3,923 ± 4,939 |  |
|  | **CCC** |  |  |  |  |  | **n=113 (4%)** |  | **n=33 (17%)** |  |
|  | Cost |  | $585 ± 7,264 |  | $4,152 ± 15,791 |  | $15,957 ± 34,690 |  | $23,906 ± 31,398 |  |
|  | **Long Term Care** | | |  |  |  | **n=70***^a^* **(2.3%)** |  | **n=3***^a^* **(1.6%)** |  |
|  | Visits (no.) |  | 0.1 ± 0.9 |  | 0.1 ± 0.8 |  | 4.6 ± 3.5 |  | … |  |
|  | LOS (days) |  | 4.8 ± 37.6 |  | 3.5 ± 30.6 |  | 212.4 ± 135.3 |  | … |  |
|  | Cost |  | $630 ± 4,987 |  | $450 ± 3,846 |  | $27,713 ± 18,677 |  | … |  |
| *^a^*mid-point of suppressed data range, n=$\pm$2.  Abbreviations: Amb., ambulatory; CCC, Complex Continuing Care; Hosp., hospital; LOS, length of stay; LTC, MH, Mental Health ; NDFP, New Drug Funding Program; ODB, Ontario Drug Benefit; OHIP, Ontario Health Insurance Plan; Rehab., rehabilitation; SDS, Same Day Surgery; TNBC, triple negative breast cancer. | | | | | | | | | | |
